# Genomics Reveals the Metabolic Potential and Functions in the Redistribution of Dissolved Organic Matter in Marine Environments of the Genus *Thalassotalea*

**DOI:** 10.3390/microorganisms8091412

**Published:** 2020-09-14

**Authors:** Minji Kim, In-Tae Cha, Ki-Eun Lee, Eun-Young Lee, Soo-Je Park

**Affiliations:** 1 Department of Biology, Jeju National University, 102 Jejudaehak-ro, Jeju 63243, Korea; mminji.k96@gmail.com; 2 Microorganism Resources Division, National Institute of Biological Resources, Incheon 22689, Korea; itcha@korea.kr (I.-T.C.); leege7@korea.kr (K.-E.L.); 3 Exhibition & Education Division, National Institute of Biological Resources, Incheon 22689, Korea; eylee1@korea.kr

**Keywords:** *Thalassotalea*, genomics, carbohydrate degradation, marine sediment, redistribution, nutrient cycling

## Abstract

Members of the bacterial genus *Thalassotalea* have been isolated recently from various marine environments, including marine invertebrates. A metagenomic study of the Deepwater Horizon oil plume has identified genes involved in aromatic hydrocarbon degradation in the *Thalassotalea* genome, shedding light on its potential role in the degradation of crude oils. However, the genomic traits of the genus are not well-characterized, despite the ability of the species to degrade complex natural compounds, such as agar, gelatin, chitin, or starch. Here, we obtained a complete genome of a new member of the genus, designated PS06, isolated from marine sediments containing dead marine benthic macroalgae. Unexpectedly, strain PS06 was unable to grow using most carbohydrates as sole carbon sources, which is consistent with the finding of few ABC transporters in the PS06 genome. A comparative analysis of 12 *Thalassotalea* genomes provided insights into their metabolic potential (e.g., microaerobic respiration and carbohydrate utilization) and evolutionary stability [including a low abundance of clustered regularly interspaced short palindromic repeats (CRISPR) loci and prophages]. The diversity and frequency of genes encoding extracellular enzymes for carbohydrate metabolism in the 12 genomes suggest that members of *Thalassotalea* contribute to nutrient cycling by the redistribution of dissolved organic matter in marine environments. Our study improves our understanding of the ecological and genomic properties of the genus *Thalassotalea*.

## 1. Introduction

The genus *Thalassotalea,* belonging to the family Colwelliaceae in the class Gammaproteobacteria, was first described by Zhang et al. [1]; the type strain *Thalassotalea ganghwensis* was isolated from the gill of a cultured flounder and was recently emended along with the reclassification of strains in the genus [2,3]. At the time of writing, the *Thalassotalea* genus includes 19 species with validly published names (https://lpsn.dsmz.de/search?word=Thalassotalea). Most *Thalassotalea* strains are Gram-stain-negative, aerobic, catalase- and oxidase-positive, rod-shaped, motile with a single polar flagellum or non-motile, and chemoheterotrophic [4]. Members of *Thalassotalea* have been isolated from various environments, including marine invertebrates (e.g., corals or oysters). In addition, some strains can grow at low temperatures (i.e., 4 °C) or in high-salt conditions (up to NaCl 9%, *w*/*v*) (see Data Set 1). A recent metagenomic analysis has suggested that *Thalassotalea* spp. contribute to the biodegradation of hydrocarbons, as important components of the microbial consortium from the Deepwater Horizon oil plume [5]. Although these species have a wide range of metabolic functions and growth conditions (including pH, temperature, and salt conditions), few studies have evaluated their genomic and metabolic traits. One study has shown that *Thalassotalea* sp. strain ND16A may have the ability to degrade oil based on a genomic analysis [6]. Additionally, genome analyses have suggested that *Thalassotalea* spp. have an advantage with respect to adaptation to habitat changes or evolutionary challenges [7,8,9], and this is supported by the numbers of RNA genes (i.e., rRNA and tRNA) in the genomes [10,11]. Taken together, these results support the hypothesis that *Thalassotalea* spp. have metabolic flexibility or the ability to adapt in marine environments; however, these previous findings are based on a small number of genomes.

*Thalassotalea piscium*, *Thalassotalea euphylliae*, *Thalassotalea crassostreae*, *Thalassotalea coralli*, and *Thalassotalea loyana* (reclassified from the genus *Thalassomonas*) have been isolated from marine invertebrates [1,12,13,14,15]. Marine invertebrates have been known as an important habitat for microbial diversity and abundance including symbionts (reviewed in [16]. In fact, this complexity for microbial communities has made it difficult to understand their metabolic-functions and interactions between the microbiota or their host (i.e., marine invertebrate). In addition, little is known about their genomic traits (with only five publicly available genomes) or the divergence between taxa with distinct lifestyles, such as associations with marine invertebrates. However, recent advances in sequencing technology, analysis tools, and the availability of complete or draft genomes, including metagenome-assembled genomes (MAGs) (e.g., Genome Taxonomy Database, GTDB) [17], have made it possible to gain insight into microbial niche adaptation, life strategies in various habitats, and bacterial taxonomy based on genome data [18,19]. In this study, to uncover the gene repertoires involved in metabolic functions and address their ecological adaptation in marine habitat, we obtained a complete high-quality genome sequence of a species in the genus *Thalassotalea*, designated PS06, isolated from costal marine sediment with dead marine benthic macroalgae, and performed a comprehensive comparative analysis with 11 additional genomes belonging to the genus *Thalassotalea*. In addition, some metabolic potentials predicted by genome analysis of the strain PS06 were demonstrated by physiological studies (e.g., utilization or degradation for substrate, and optimal growth conditions).

## 2. Materials and Methods

### 2.1. Sample Collection, Isolation, and Physiological Test

Strain PS06 was isolated from a marine sediment sample with various dead marine benthic macroalgae collected off the coast of Jeju Island, South Korea (N 33°31′39″, E 126°35′15″). To isolate microorganisms, debris was removed, and samples were mixed with artificial seawater (ASW) medium to prepare a slurry. The basic components of ASW medium per liter (L) were as follows: 4.66 g MgCl_2_∙6H_2_O, 0.67 g KCl, 0.2 g KH_2_PO_4_, 24.6 g NaCl, 6.29 g MgSO_4_∙7H_2_O, and 1.36 g CaCl_2_∙2H_2_O adjusted to 7.5 by using 1 N NaOH or HCl. The slurry sample was diluted 10-fold to 10^−6^, and 100 μL aliquots were inoculated on marine agar 2216 (MA; BD Difco, Franklin Lakes, NJ, USA). The plates were then incubated under dark and aerobic culture conditions at 30 °C for 10 days. By the naked eye, small single colonies were transferred to new MA plates and incubated again under the same conditions. Finally, one isolate, designated PS06, was cultured routinely on MA medium at 30 °C and preserved as a suspension in marine broth medium with glycerol (30%, *v*/*v*) at −80 °C.

For phenotypic characterization, strain PS06 was grown routinely on marine 2216 agar (Difco, MA) at 30 °C to the mid-exponential phase, unless indicated otherwise. The range and optimal temperature for growth were determined by incubation on MA at 5 °C to 45 °C (intervals of 5 °C). The range and optimal pH for growth were determined at pH 4.0–10.5 (at intervals of 0.5 pH units) on marine 2216 broth (Difco, MB) using the following buffering system: homopiperazine-1,4-bis-2-ethanesulfonic acid (pH 4.0–5.0), 2-(*N*-morpholino) ethanesulfonic acid (pH 5.5–6.5), 1,3-bis[tris (hydroxymethyl)methylamino]propane (pH 7.0–8.5), and 3-(cyclohexylamino)-1-propanesulfonic acid (pH 9.0–10.5). To estimate salinity tolerance, the strain was grown in modified ASW medium supplemented with yeast extract 0.5% (*w*/*v*) and peptone 0.5% (*w*/*v*) containing 0–10% (*w*/*v*) NaCl (at intervals of 1%). Catalase activity was determined by the production of oxygen bubbles after mixing cells with 3% (*v*/*v*) H_2_O_2_. An oxidase test was performed using 1% (*w*/*v*) tetramethyl-*p*-phenylenediamine (Merck, Kenilworth, NJ, USA). Anaerobic and microaerobic growth were investigated after 2 weeks of incubation using the GasPak^TM^ EZ Anaerobe and Campy Pouch System with Indicator (BD, Difco), respectively. In addition, an anaerobic respiration test was performed using a MA containing nitrate (3 mM) or nitrite (1 mM). The hydrolysis of starch (0.5%, *w*/*v*), skim milk (0.2%, *w*/*v*), gelatin (12%, *w*/*v*), and Tweens 20, 40, 60, and 80 (1.0%, *v*/*v*) was determined based on the formation of clear zones around colonies after applying suitable solutions, according to the method described by Reddy et al. [20]. DNA degradation was tested using DNase Test Agar (BD Difco) modified with up to 3.0% NaCl. Biochemical tests were carried out using the API ZYM Test Strip (bioMérieux, Marcy-l’Étoile, France) and GEN III MicroPlate^TM^ (Biolog, Hayward, CA, USA) with bacterial suspensions in the salt-adjusted medium.

### 2.2. Phylogenetic Analysis

To determine the phylogenic position of the newly isolated strain PS06, genomic DNA (gDNA) was extracted using a commercial genomic DNA extraction kit (GeneAll Biotechnology Co., Ltd., Seoul, Korea) according to the manufacturer’s instructions. The 16S rRNA gene was amplified by polymerase chain reaction (PCR) using the universal bacterial primer set 27F (5′-AGAGTTTGATCMTGGCTCAG-3′) and 1492R (5′-TACGGYTACCTTGTTACGACTT-3′) [21]. The amplification conditions were as follows: pre-incubation at 95 °C for 5 min; followed by 30 cycles at 95 °C for 30 s, 55 °C for 30 s, and 72 °C for 90 s; and a final extension at 72 °C for 7 min. The PCR product was purified using a PCR Purification Kit according to the manufacturer’s instructions (GeneAll Biotechnology Co., Ltd., Seoul, Korea). The 16S rRNA gene was sequenced using primers 27F, 518F, 800R, and 1492R, as described previously [21,22]. Finally, the 16S rRNA gene sequence of strain PS06 (approximately 1,450 bp) was assembled using SeqMan (DNASTAR) and compared with the sequences of related taxa obtained from the GenBank database (www.ncbi.nlm.nih.gov) and the EzBioCloud server (https://www.ezbiocloud.net). The sequences were edited using BioEdit and aligned using Clustal_X [23,24]. Evolutionary distances were calculated using the Kimura two-parameter model [25]. The maximum likelihood method [26] was used to reconstruct phylogenetic trees using MEGA X [27], and bootstrap values were obtained based on 1000 replicates [28].

### 2.3. Genome Sequencing, Assembly, and Annotation

For whole-genome sequencing of strain PS06, gDNA was prepared using the FastDNA SPIN Kit (MP Biomedical, Santa Ana, CA, USA) according to the manufacturer’s instructions. The genome was sequenced in one single molecule, real-time (SMRT) cell on a PacBio RS II sequencing instrument (10 kb library, approximately 101-fold coverage). The de novo genome assembly was obtained using PacBio SMRT Analysis (version 2.3) and the HGAP2 hierarchical genome assembly process with default settings.

After assembly, the putative coding sequences (CDSs) for strain PS06 and other public genomes were predicted using Prodigal [29]. Protein sequences were annotated using the best basic local alignment search tool (BLAST) hit against the NCBI NR database, and tRNAs were identified using tRNAscan-SE [30]. A similarity analysis was performed using Clusters of Orthologous Groups (COGs), TIGRfam, Pfam, and Kyoto Encyclopedia of Genes and Genomes (KEGG) database references, as described previously [31,32,33]. Secondary metabolite biosynthesis and carbohydrate utilization genes were identified using antiSMASH (version 5.1.0) [34] and dbCAN [35], respectively, and genome completeness was assessed using CheckM [36]. TonB-dependent transporters (TBDT) were classified based on the classification of Tang et al. [37].

Tandem repeats (TRs) were identified using the TRDB (Tandem Repeats Database) [38]. Orthologous gene clusters and prophage sequences of the isolate and reference genomes were identified using OrthoVenn2 [39] and PHASTER [40], respectively.

### 2.4. Genomic and Phylogenomic Analyses

Publicly available complete or draft genomes or MAGs belonging to the *Thalassotalea* (n = 11) were downloaded from the Genome Taxonomy Database (GTDB, https://gtdb.ecogenomic.org/). More detailed information regarding the isolation source, physiological characteristics, accession numbers, and sequence statistics for all genomes used in this study is provided in Table S1.

We performed phylogenomic analyses of the genomes of strain PS06 and related taxa (n = 13, including those in the closely related genus *Thalassomonas*). Single-copy marker genes (n = 161; Table S2) were used to infer a phylogeny based on the concatenated protein sequence, as previously described [32]. Genomes were included in the phylogenomic analysis if they harbored at least 20% (38 markers) of the phylogenetic markers. Individual homologous proteins were aligned using PRANK (with default parameters) [41], trimmed using BMGE (−t AA −g 0.2 −b 1 −m BLOSUM65) [42], concatenated, and subjected to SR4 amino acid recoding [43]. A maximum likelihood tree was generated using IQ-TREE [44] with the best-fitting substitution model (identified as GTR+F+R6 using ModelFinder) [45], and ultrafast bootstrapping (−bb 1000 −alrt 1000) [46]. Average nucleotide identity (ANI) and average amino acid identity (AAI) for all genomes were calculated as previously described [47,48].

### 2.5. Data Availability

Strain PS06 was deposited in the Korean Culture Center of Microorganisms (KCCM) under the accession number 43300. The complete genome sequence of strain PS06 was deposited in the EMBL/GenBank under the accession number CP041638.

## 3. Results and Discussion

### 3.1. General Genomic Features

The genome of strain PS06 consisted of a single circular chromosome of about 3.84 Mbp with a mean GC content of 44.2%. In total, 3211 CDSs, accounting for 86.2% of the genome, were predicted, in addition to 81 tRNAs and 19 rRNAs. The genome completeness was 97.3% with 1.6% contamination and 0.0% strain heterogeneity, as estimated using CheckM (Table S1). Among CDSs, 2519 were assigned to COG functional categories. The COG categories [J] Translation, ribosomal structure and biogenesis (222 CDSs, 8.8%), followed by [R] General function prediction only (218 CDSs, 8.7%), [E] Amino acid transport and metabolism (217 CDSs, 8.6%), [M] Cell wall/membrane/envelope biogenesis (185 CDSs, 7.3%), [C] Energy production and conversion (182 CDSs, 7.2%), and [S] Function unknown (161 CDSs, 6.4%), accounted for 47.0% of the overall CDSs (Figure S1). Potential metabolic functions (e.g., biodegradation and homeostasis) of strain PS06 and other genomes were further analyzed using various functional gene databases, including KEGG (Table S3).

### 3.2. Phylogenetic and Phylogenomic Analyses

In a 16S rRNA gene sequence analysis, strain PS06 clustered with species belonging to the genus *Thalassotalea* (Figure S2). In a comparison of the 16S rRNA gene sequence extracted from the whole-genome sequence and that obtained by Sanger sequencing, no differences were apparent.

**Figure 1:**
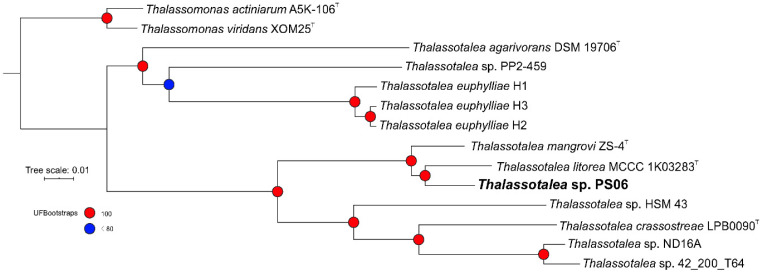
Phylogenomic tree based on draft or complete genomes of PS06, members of the genus *Thalassotalea*, and two *Thalassomonas* spp. Maximum likelihood phylogenies were inferred from 14 genomes. The PS06 genome is highlighted in bold. The strength of support for internal nodes was assessed by bootstrap resampling, and values are shown as colored circles (see legend). Midpoint rooting was used.

According to information available on the EzBioCloud server, strain PS06 was most closely related to *Thalassotalea litorea* HMF4135 (98.3% 16S rRNA gene sequence similarity), *Thalassotalea ponticola* GJSW-36 (95.9%), *Thalassotalea agarivorans* TMA1 (95.4%), and *Thalassotalea fusca* G-MB1 (94.9%). In addition, the phylogenetic tree based on 16S rRNA gene sequences showed that *T*. *litorea*, *T*. *ponticola*, *T*. *crassostreae*, and strain PS06 formed a cluster that was well separated from other strains in the genus *Thalassotalea*, and these strains were isolated from South Korea [3,12,49]. Based on the phylogenomic tree (Figure 1), strain PS06 clustered with *T*. *litorea* and *Thalassotalea mangrovi* [4], and three genomes (*T. euphylliae* H1, H2, and H3) isolated from corals in Hawaii, USA, formed a single cluster [10,11,14]. This result indicated that some species of the genus *Thalassotalea* might have more genomic relatedness than sequence similarity in their 16S rRNA genes and occupy the same ecological riches based on evolutionary relationships [50,51].

**Figure 2:**
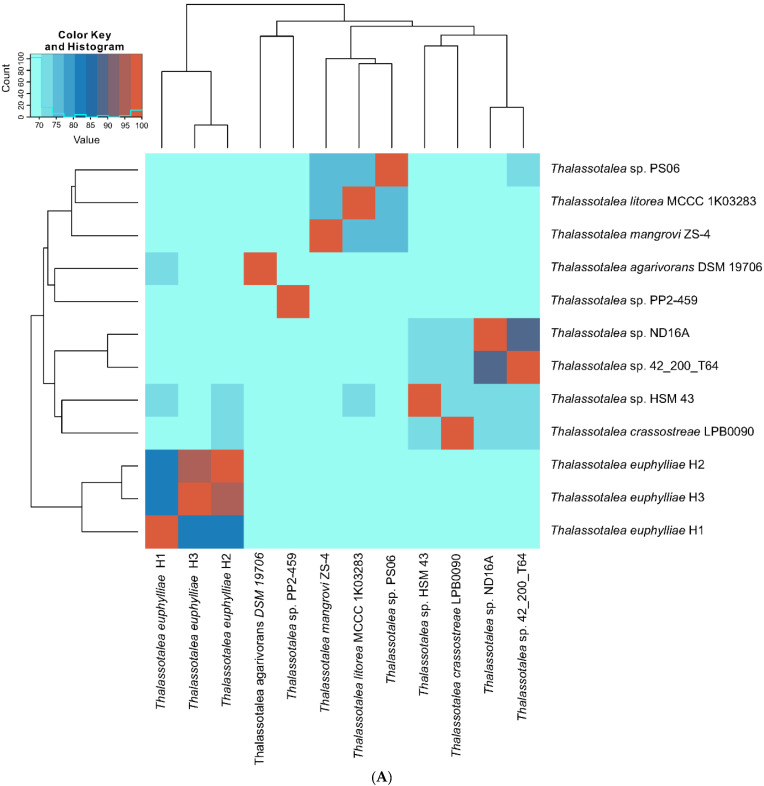

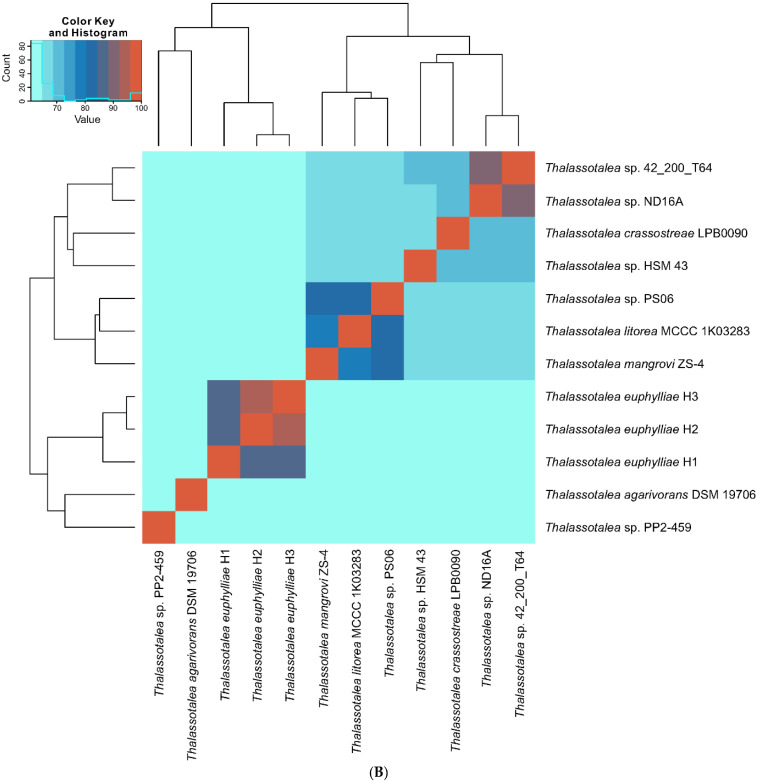
Heatmap of genomic relatedness between PS06 and other members of the genus *Thalassotalea*. The average nucleotide identity (ANI; (**A**)) and average amino acid identity (AAI; (**B**)) between pairs of genomes were calculated.

Genome-wide ANI and AAI comparisons further delineated strain PS06 into a single cluster (Figure 2), consistent with the phylogenomic tree. ANI and AAI values for the comparison between PS06 and the most closely related described species, *T*. *litorea*, were 76.7% and 82.1%, respectively. In accordance with the guidelines described by Konstantinidis et al. (30), these ANI and AAI values clearly support the designation of strain PS06 as a novel candidate species in the genus *Thalassotalea*.

### 3.3. Comparative Genome Analysis of Thalassotalea Strains

We analyzed 12 genomes of members of the genus *Thalassotalea*, including four validated type strains (Table S1). Seven genomes were obtained from public databases, and strain PS06 was newly sequenced in this study. Most strains were isolated from marine environments, including marine invertebrates (e.g., *Crassostrea gigas*, *Ruditapes decussatus*, or corals). One MAG, *Thalassotalea* sp. 42_200_T64, was obtained from a late-stage hydrocarbon degradation mimic-enriched culture in a deep-sea oil plume (Deepwater Horizon) [5]. These 12 genomes were nearly complete, with an average completeness of 97.8%, contamination of 1.04%, and strain heterogeneity of 0.6% (see Table S1). The expected genome sizes ranged from 3.37 Mb for *T. agarivorans* DSM 19706 to 4.75 Mb for *T. euphylliae* H1. Only two strains (*T. crassostreae* LPB0090 and *Thalassotalea* sp. PP2-459) isolated from calm areas had low GC contents (38.8% and 38.3%, respectively), whereas other genomes had average GC contents of 43.3% (41.6–45.9%). This high variation in genome size and GC content suggests that the members of the genus *Thalassotalea* experience different environmental conditions and have distinct physiological abilities [52,53], despite the similar functional profiles based on COG categories (Figure S1).

In a genome-wide comparison of orthologous genes across 12 genomes of the genus *Thalassotalea,* we detected 5877 clusters, 4560 orthologous clusters (containing at least two species), and 1317 single-copy gene clusters. Contrary to a previous hypothesis (i.e., different strategies for environmental adaptation), most orthologous genes were shared among all genomes in the analysis. Additionally, 44 unique CDSs in the strain PS06 genome were grouped into four clusters: response to oxidative stress, DNA replication, nucleotide binding, and transferase activity for glycosyl groups. These results indicated that each strain in the genus has a unique survival strategy, beyond the observed physiological characteristics.

### 3.4. Central Metabolism, Substrate Utilizations, and Respiration

The PS06 genome contained complete central metabolic pathways (Table S3): glycolysis (Embden–Meyerhof pathway), gluconeogenesis, pyruvate oxidation, tricarboxylic acid (TCA) cycle, pentose phosphate pathway, PRPP biosynthesis, and Entner–Doudoroff pathway. In addition, gene repertoires for glycogen biosynthesis and degradation in the genome were complete. Based on a KEGG analysis, we also deduced that strain PS06 has alternative pathways for carbon, despite some missing genes involved in the glyoxylate cycle, anabolic pathway for C_2_ compounds, malonate semialdehyde pathway (aldehyde oxidation) [54], and d-galactonate degradation (i.e., DeLey–Doudoroff pathway) [55]. Genes in the DeLey–Doudoroff pathway have been identified in algal polysaccharide-degrading marine microorganisms [56,57]. Although strain PS06 did not use d-galactose as a growth substrate, it might be able to use it for ascorbate biosynthesis [58,59].

To estimate substrate utilization, we screened transporters in the genome. Unexpectedly, although the strain has been recognized as a complete chemoheterotroph, the genome encoded few transporters of organic matter, such as lipoprotein (LolCED), phosphate (PstSCAB), capsular polysaccharide (KpsEMT), lipopolysaccharide (LptFGB), phospholipid (MlaCDEBF), and defensin (SapABCDF). The PS06 genome harbored transporters for inorganic matter, including molybdate (ModAB), iron (III) (AfuABC), sodium ion (NatBA), and heme [ccmDCBA (exporter)]. Based on the prediction for substrate utilization based on genomic traits, we evaluated physiological characteristics, as described previously [22]. Strain PS06 was able to utilize a limited number of substrates, including dextrin, d-trehalose, d-cellobiolose, and d-mannose (Table S4), which are related to some transport systems identified in the genome.

With respect to oxidative phosphorylation (i.e., ATP synthesis), the PS06 genome harbored genes necessary for aerobic respiration, including those for complex I (NADH:ubiquinone oxidoreductase), complex II (succinate dehydrogenase), and complex III (cytochrome *bc*1 complex). Genes encoding F-type ATPases were identified. Two types of terminal cytochrome oxidases were identified, complex IV: *c* type (CoxDCAB) and *cbb*_3_ type (CooNOQP). Additionally, the PS06 genome contained genes for other respiratory pathways. We found complete gene repertoires for assimilatory nitrate reduction (to ammonia, NapABCDEF and nirBD) and assimilatory sulfate reduction (to sulfide, cysNDCHJI) in the genome. The PS06 genome had a nitrate and nitrite transporter (NrtABCD). Also, only NasAB-encoding genes involved in assimilatory nitrate reduction were identified. However, genes coding for the dissimilatory nitrate reduction (denitrification and dissimilatory nitrate reduction to ammonia) were not identified in the PS06 genome, suggesting strain PS06 might be unable to be grown under anaerobic condition with inorganic electron acceptors (i.e., nitrate or nitrite) [60]. Moreover, anaerobic growth of the strain PS06 was not observed. In agreement with this, strain PS06 is likely capable of growth under high oxygen concentrations as well as microaerobic culture condition.

### 3.5. Metabolic Potentials

The PS06 genome contained few genes encoding transporters of cationic peptides, lipopolysaccharides, capsular polysaccharides, lipoproteins, vitamin B_12_, and phospholipids. Additionally, we found transporters for dissolved organic matter (DOM, cluster 3090 and 427) and siderophore/vitamins (cluster 410) in the PS06 genome based on the TBDT database [37], as well as in other genomes analyzed in this study (Table 1 and Data Set 2). These results suggest that members of the genus *Thalassotalea* play roles in marine nutrient cycling by the redistribution of DOM or inorganic compounds.

Moreover, strain PS06 can only grow on a complex medium supplied with yeast extract or peptone as a growth factor [61,62,63]. Consistent with this, the PS06 genome harbored few genes related to vitamin metabolism (i.e., biotin and tetrahydrofolate biosynthesis).

Two copies of the gene encoding poly-3-hydroxybutyrate (PHB) depolymerase were identified in the PS06 genome, despite the lack of genes involved in PHB biosynthesis. These results indicate that strain PS06 might contribute to the biodegradation of bio-polyester materials consisting of polyhydroxyalkanoic acids and utilize exogenous carbon or energy sources from extracellular PHB compounds [64,65].

As a branched polypeptide, cyanophycin (cyanophycin granule polypeptide, CGP) is an insoluble-reserve material of cyanobacteria [66] and non-cyanobacteria [67,68]. Interestingly, genes for type 1 glutamine amidotransferase (GATase1)-like domain and cyanophycinase (CphB) have been identified in the genome of the strain PS06 [69]. This result indicates that strain PS06 can use extracellular CGP as a valuable source of nitrogen, carbon, and energy [70,71], which would provide it with an ecological advantage over other bacteria in its habitat.

### 3.6. Repertoire of Carbohydrate-Active Enzymes (CAZymes) and Degrading Activities of the Genus Thalassotalea

The PS06 genome harbored various genes involved in carbohydrate metabolism, including genes encoding carbohydrate-active enzymes (CAZymes). The identified CAZymes provide insight into their lifestyles and potential applications in biomedicine or biotechnology [72,73]. CAZymes have been classified by following modules: glycoside hydrolases (GHs), glycosyltransferases (GTs), polysaccharide lyases (PLs), carbohydrate esterases (CEs), auxiliary activities (AAs), and carbohydrate-binding modules (CBMs). Also, bacterial CAZymes have been characterized for synthesis and degradation of polysaccharides as a rich source of bioactive compounds in algae including seaweeds and recognized as an eco-friendly enzyme for improving the yield and quality for derivatives of algal polysaccharides [73,74]. Although a number of marine heterotrophic bacteria are involved in the degradation of various biopolymers including, polysaccharide degradation, little is known about the gene repertories of hydrolytic systems in the genus *Thalassotalea*. The *Thalassotalea* genomes, including PS06, had genes encoding CAZymes in various functional classes (Table 2 and Data Set 2), as well as TonB-dependent transporters (TBDTs). In particular, GHs, GTs, and CEs were the most abundant CAZymes in all taxa analyzed in this study, suggesting that the members of the genus *Thalassotalea* have the ability to degrade the surface matrix of hosts (e.g., sponges and algae) [75]. Sixty-nine GH family genes (classified by DIAMOND with the removal of other families) were identified in all genomes. Most *Thalassotalea* genomes shared GH family genes (43 of 69 classes, n < 2 overlapping GH genes between genomes), indicating a lack of positive selection [76]. Nevertheless, the identification of unique GH families suggests specific adaptation for carbohydrate utilization and degradation according to habitats [77].

Genes encoding PLs were found in some genomes, including that of strain PS06, although only four PL family members were identified (i.e., PL1, 6, 7, or 17). PLs are a class of cleavage enzymes for uronic acid-containing polysaccharides (e.g., alginate) by a β-elimination mechanism, producing unsaturated products [78]. Therefore, the PLs of the *Thalassotalea* spp. could be used as highly active and specific enzymes for energy and chemical production via glycan saccharification [79]. Taken together, these enzymes for polysaccharide degradation of the *Thalassotalea* spp. might receive exceptionally attention to their biotechnological or medical potentials.

In addition, members of the genus *Thalassotalea* have the ability to hydrolyze polymer materials, such as agar, starch, casein, gelatin, DNA, Tweens, or chitin (Data Set 1). We confirmed that PS06 hydrolyzes gelatin, starch, and Tween 40 and 80, and shows positive activity for esterase (C4), esterase lipase (C8), lipase (C14), valine arylamidase, trypsin, acid phosphatase, Naphtol-AS-BI-phosphohydrolase, β-glucosidase, and *N*-acetyl-β-glucosaminidase. These results were supported by the identification of genes involved in carbohydrate assimilation and peptidase in the 12 genomes included in the study (Table 2 and Data Set 2). Therefore, these results provide further evidence that strain PS06 acts as a recycler for exogenous polymers, including detrital proteins, in marine environments. Additionally, genes involved in the twin-arginine translocation (Tat) system (*tatABC*) for protein translocation across the cytoplasmic membrane were clearly identified in the genome [80,81].

### 3.7. Environmental Stress and Adaptation

We determined the growth conditions according to previously described methods [22]. We found that strain PS06 has general mesophilic features, with growth temperatures ranging from 5 °C to 40 °C (optimal, 30 °C). Strain PS06 was an alkaliphile and moderate halophile based on growth at pH 7 to 10 (optimal, pH 9) and 1% to 10% *w*/*v* NaCl (optimal, 5%). As we mentioned above, the strain PS06 grew under aerobic and microaerobic culture conditions. Accordingly, we screened the genome for genes related to reactive oxygen species (ROS) stress, pH homeostasis, and osmoregulation.

The activity for catalase and oxidase was observed in strain PS06. In addition, enzymes involved in ROS stress (e.g., superoxide dismutase, glutathione peroxidase, putative iron-dependent peroxidase, and thiol peroxidase) were identified its genome. Generally, halophiles and/or alkaliphiles have genes encoding a vacuolar ATP synthase (V_0_V_1_-type, Ntp), multisubunit electrogenic sodium/proton antiporter (Mrp), compatible solutes (e.g., trehalose, ectoine, glycine betaine), osmoprotectant transport system (Opu), or cation/proton antiporter (CPA) superfamily (reviewed in [82]). Multisubunit Na^+^/H^+^ (Mnh), Ca^2+^/Na^+^, and Na^+^(K^+^)/H^+^ (Nha) antiporter genes were identified in the PS06 genome. Surprisingly, although this organism is a strict halophile, no genes involved in the synthesis of osmoprotection compounds (i.e., compatible solutes; trehalose, ectoine, and betaine) or osmoprotectant transporter genes (OpuCBDA) have been detected [83].

Additionally, the PS06 genome contained the complete set of genes for isoprenoid biosynthesis via the non-mevalonate pathway, even though full sets of genes in the pathway have only been reported in a few bacteria (reviewed in [84]). However, we did not find putative genes for squalene synthase (Hpn), which functions in the formation of squalene from its precursors [85]. These results indicated that strain PS06 might lack membrane properties for adaptation to haloalkaline environments, although the strain can grow in high-salt (up to 10%, *w*/*v*) or pH (up to 10) culture conditions. Nevertheless, the negatively charged cell membrane may be an alternative strategy for the survival of strain PS06 in haloalkaline environments [86,87].

Gene sets for flagellar biosynthesis and bacterial chemotaxis were lacking in the PS06 genome, whereas genes for Pil components essential for type IV pilus (Trp) assembly, tight adherence (Tad), and Flp (fimbrial low-molecular-weight protein) were found [88,89]. In addition, the genome contained genes for capsular polysaccharide biosynthesis (CPS), capsule polysaccharide export protein (*kpsESC*), and sugar phosphate isomerase (*gutQ*), involved in capsule formation and the type II secretion system (e.g., cell wall-degrading enzyme secretion), suggesting that PS06 has an advantage against competitors (e.g., prey) [90] or in harsh environments [91]. Additionally, using antiSMASH (version 5.1.0) [34] we attempted to identify putative antibiotic biosynthesis gene clusters. The PS06 genome had no genes related to secondary metabolite biosynthesis, whereas other genomes had a few secondary metabolite clusters, such as resorcinol, bacteriocin, hserlactone (homoserine lactone), ectoine, thiopeptide, or NRPS (non-ribosomal peptide synthetase cluster)-like (Table S5). Taken together, *Thalassotalea* spp. might have various strategies to cope with competitors in their habitats.

We further evaluated correlations between genomic traits and habitats or hosts. Generally, intergenic TRs are associated with various bacterial phenotypes (e.g., cell surface structure, motility, or restriction-modification systems) [92] related to survival in changing environments by genome flexibility. Most *Thalassotalea* genomes have hundreds of TRs (range 245 to 384, average 301), other than *T*. *agarivorans* (n = 80) and *Thalassotalea* sp. PP2-459 (n = 70). Although it is difficult to compare across genera or habitats, members of the genus *Thalassotalea* might have an advantage with respect to adaptation to environmental stresses. With respect to defense mechanisms against viral predation in the genomes, few clustered regularly interspaced short palindromic repeats (CRISPR) candidates and spacers were identified in the 11 genomes (Table S6). On the other hand, a number of CRISPR loci (n = 6) with spacers (n = 102) have been reported in the *T*. *euphylliae* H1 genome. The CRIPSR-Cas system has been identified in about half of all bacterial genomes as an adaptive immune system [93]. In addition, an array of repetitive and unique sequences (referred to as repeats and spacers, respectively) have evolved in their genetic relatedness. As mentioned above, except for the *T*. *euphylliae* H1 genome, most *Thalassotalea* genomes (n = 11) have a few CRISPR and spacers. This suggests that members of the genus *Thalassotalea* might be less subject to evolutionary (i.e., it is genomically stable) or phage infection stresses in their ecological niches [93,94,95].

To estimate the frequency of genomic rearrangements in *Thalassotalea* spp., we analyzed prophage regions [33]. Most genomes had incomplete or no prophages, whereas two questionable prophage regions were identified in the *T*. *euphylliae* H1 genome (Table S7). Taken together, the long-term evolutionary pressure on genomes of *Thalassotalea* spp. might be low, despite the lack of an efficient defense mechanism. This hypothesis should be evaluated using more isolates and genomes in the future.

## 4. Conclusions

Physiological and genomic analyses indicated that strain PS06 is capable of surviving under haloalkaline environments by alternative strategies in various marine environments. This inference is supported by the detection of TRs and genes encoding extracellular enzymes. In fact, the metabolic potential of PS06 suggests that the strain is an obligate chemoheterotroph (requiring complex organic compounds as an energy source, e.g., peptone or yeast extract), although it also harbors a limited set of genes for substrate transporters. In addition, *Thalassotalea* spp. including strain PS06, can degrade macroalgal-derived polysaccharides and contribute to nutrient cycling in marine environments. In addition, the ability for polysaccharide degradation of the genus *Thalassotalea* suggest an application to biotechnology or medicine. Finally, our results demonstrate that members of the genus *Thalassotalea* might have diverse metabolic pathways and ecological strategies in haloalkaline ecosystems.

## Figures and Tables

**Table 1:** Summary of genes encoding TonB-dependent transporters (TBDTs) in *Thalassotalea* genomes. TBDTs are classified based on Tang et al. [37].

Function Categories	Lek Cluster Number	Substrates	*Thalassotalea* sp. ND16A	*Thalassotalea crassostreae* LPB0090	*Thalassotalea* sp. PP2-459	*Thalassotalea* sp. 42_200_T64	*Thalassotalea euphylliae* H1	*Thalassotalea euphylliae* H3	*Thalassotalea euphylliae* H2	*Thalassotalea* sp. HSM 43	*Thalassotalea mangrovi* ZS-4	*Thalassotalea litorea* MCCC 1K03283	*Thalassotalea* sp. PS06	*Thalassotalea agarivorans* DSM 19706
Group I: Dissolved organic matter transporters	Cluster_3090	Chito-oligosaccharides, phytate, maltodextrin, maltose, chitin, xylan, xylose, pectin	13	10	14	7	7	4	6	10	9	13	8	11
	Cluster_720	Digested proteins, starch/malto-oligo-saccharides, chondroitin sulfate/hyaluronic acid	-	-	-	-	-	-	-	-	-	-	-	-
	Cluster_427	Arabinose	7	4	7	5	10	7	7	6	9	5	7	4
	Cluster_952	Sucrose	-	-	1	-	-	-	-	-	-	-	-	-
Group II: Siderophores/Vitamins transporters	Cluster_3303	Ferric-citrate	-	-	1	-	-	1	1	-	-	1	-	-
	Cluster_410	Aerobactin, alcaligin, anguibactin, catecholates, chrysobactin, coprogen, ferrioxamine B, rhodoturolic acid, desferrioxamine, ferric malleobactin, ferric ornibactin, ferrichrome, hexylsulfate, pseudobactin A, pseudobactin M114, pyochelin, pyoverdine, rhizobactin 1021, thiamin, vibriobactin, yersiniabactin	7	8	10	4	17	16	14	9	13	10	11	8
	Cluster_973	Vitamin B12, catecholates, enterobactin, 2,3-dihydroxybenzoylserine(DHBS)	2	3	6	5	6	5	4	2	1	2	1	3
	Cluster_325	Vitamin B12	-	-	-	1	-	-	-	-	-	-	1	-
	Cluster_180	Fibronectin, thiamin	-	-	-	1	-	-	-	-	-	-	-	-
	Cluster_2835	Thiamin	-	-	-	-	1	-	-	-	-	-	-	-
Group III: Heme/Hemophores/Iron(heme)-binding transporters	Cluster_1609	Heme	-	-	2	-	1	1	1	1	1	1	1	1
	Cluster_1856	Heme	1	-	2	-	4	3	3	1	2	2	-	-
Group IV: Metal transporters	Cluster_767	Copper, Copper chelate	-	-	1	-	1	1	1	-	-	-	-	-
	Cluster_987	Nickel, Cobalt	-	-	1	-	-	-	-	-	-	-	1	-

**Table 2:** Number of genes involved in carbohydrate assimilation in the 12 genomes included in this study.

Strain	Transporters by TCDB ^a^	CAZy ^b^	Peptidases by MEROPS ^c^	TBDT ^d^
*Thalassotalea* sp. ND16A	215	117	75	30
*Thalassotalea crassostreae* LPB0090	180	138	51	25
*Thalassotalea* sp. PP2-459	206	118	80	45
*Thalassotalea* sp. 42_200_T64	180	107	58	23
*Thalassotalea euphylliae* H1	199	121	63	47
*Thalassotalea euphylliae* H3	196	112	65	38
*Thalassotalea euphylliae* H2	193	114	65	37
*Thalassotalea* sp. HSM 43	205	145	74	29
*Thalassotalea mangrovi* ZS-4	189	132	62	35
*Thalassotalea litorea* MCCC 1K03283	190	147	65	34
*Thalassotalea* sp. PS06	176	128	67	30
*Thalassotalea agarivorans* DSM 19706	171	126	65	27

^a^ BLASTp search against the Transporter Classification Database (TCDB), cutoff: protein similarity ≥ 50%, coverage ≤ 70%. ^b^ BLASTp search against the Carbohydrate-active Enzymes Database (CAZy) annotated by the three methods: HMMER, diamond, and Hotpep by dbCAN. DIAMOND: E-Value < 1e-102, hits per query (-k) = 1, HMMER: E-Value < 1e-15, coverage > 0.35, Hotpep: Frequency > 2.6, Hits > 6. ^c^ BLASTp search against the peptidase database (MEROPS). ^d^ TonB-dependent transporters (TBDT).

## Supplementary Materials

The following are available online at https://www.mdpi.com/2076-2607/8/9/1412/s1. Figure S1. Distribution of clusters of orthologous groups (COGs) for loci in PS06 and other genomes. The x-axis represents the percentage of genes in each COG category. Bars indicate COG functional classes. Figure S2. Phylogenetic tree inferred by the maximum likelihood method and Kimura 2-parameter model based on 16S rRNA gene sequences showing the phylogenetic relationships among strain PS06 and related strains, including members of the genus *Thalassotalea* and closely related taxa. The sequence of *Halomonas alkaliantarctica* CRSS (AJ564880) was used as the outgroup. The GenBank accession numbers are given in parentheses. The tree is drawn to scale, with branch lengths proportionate to the number of substitutions per site. This analysis involved 37 nucleotide sequences and 1473 total sites. Evolutionary analyses were conducted using MEGA X. Table S1. Summary statistics, including genomic completeness determined by CheckM, and detailed information about geographical locations and sites of strains included in this study. Table S2. TIGRfams markers used for the phylogenomic analysis. Table S3. Kyoto Encyclopedia of Genes and Genomes (KEGG) orthology annotation for PS06 and other genomes. Presence/absence information for listed KO numbers is shown for each individual MAG in columns F:Q (1 indicates present and 0 indicates absent). Table S4. Physiological characteristics (enzyme activity and substrate utilization) of strain PS06 determined by GEN III (Biolog) and API ZYM. Table S5. Predicted secondary metabolite clusters, as determined by antiSMASH5. All secondary metabolite clusters predicted for PS06 and other genomes. Table S6. Clustered regularly interspaced short palindromic repeat (CRISPR)-associated genes identified in PS06 and other genomes. Table S7. Questionable prophage regions identified in PS06 and other genomes.
