# Supplementary material for: Genomics Reveals the Metabolic Potential and Functions in the Redistribution of Dissolved Organic Matter in Marine Environments of the Genus Thalassotalea
Source: Microorganisms. 2020 Sep 14;8(9):1412. doi: 10.3390/microorganisms8091412 (PMC7564069; doi:10.3390/microorganisms8091412)
Supplement: Supplementary file 1 [file microorganisms-08-01412-s001.zip › Stable_figure-legends.docx]

**Supplementary table and figure legends**

**Table S1.** Summary statistics, including genomic completeness determined by CheckM, and detailed information about geographical locations and sites of strains included in this study

**Table S2.** TIGRFAMs markers used for the phylogenomic analysis

**Table S3.** KEGG Orthology annotation for PS06 and other genomes. Presence/absence information for listed KO numbers is shown for each individual MAG in columns F:Q (1 indicates present and 0 indicates absent)

**Table S4.** Physiological characteristics (enzyme activity and substrate utilization) of strain PS06 determined by GEN III (Biolog) and API ZYM

**Table S5.** Predicted secondary metabolite clusters, as determined by antiSMASH5. All secondary metabolite clusters predicted for PS06 and other genomes

**Table S6.** Clustered regularly interspaced short palindromic repeat (CRISPR)-associated genes identified in PS06 and other genomes

**Table S7.** Questionable prophage regions identified in PS06 and other genomes

**Figure S1.** Distribution of Clusters of Orthologous Groups (COGs) for loci in PS06 and other genomes. The *x*-axis represents the percentage of genes in each COG category. Bars indicate COG functional classes.

**Figure S2.** Phylogenetic tree inferred by the maximum likelihood method and Kimura 2-parameter model based on 16S rRNA gene sequences showing the phylogenetic relationships among strain PS06 and related strains, including members of the genus *Thalassotalea* and closely related taxa. The sequence of *Halomonas alkaliantarctica* CRSS (AJ564880) was used as the outgroup. The GenBank accession numbers are given in parentheses. The tree is drawn to scale, with branch lengths proportionate to the number of substitutions per site. This analysis involved 37 nucleotide sequences and 1473 total sites. Evolutionary analyses were conducted using MEGA X.
