# Supplementary figures and images for: Genomics Reveals the Metabolic Potential and Functions in the Redistribution of Dissolved Organic Matter in Marine Environments of the Genus Thalassotalea
Source: Microorganisms. 2020 Sep 14;8(9):1412. doi: 10.3390/microorganisms8091412 (PMC7564069; doi:10.3390/microorganisms8091412)

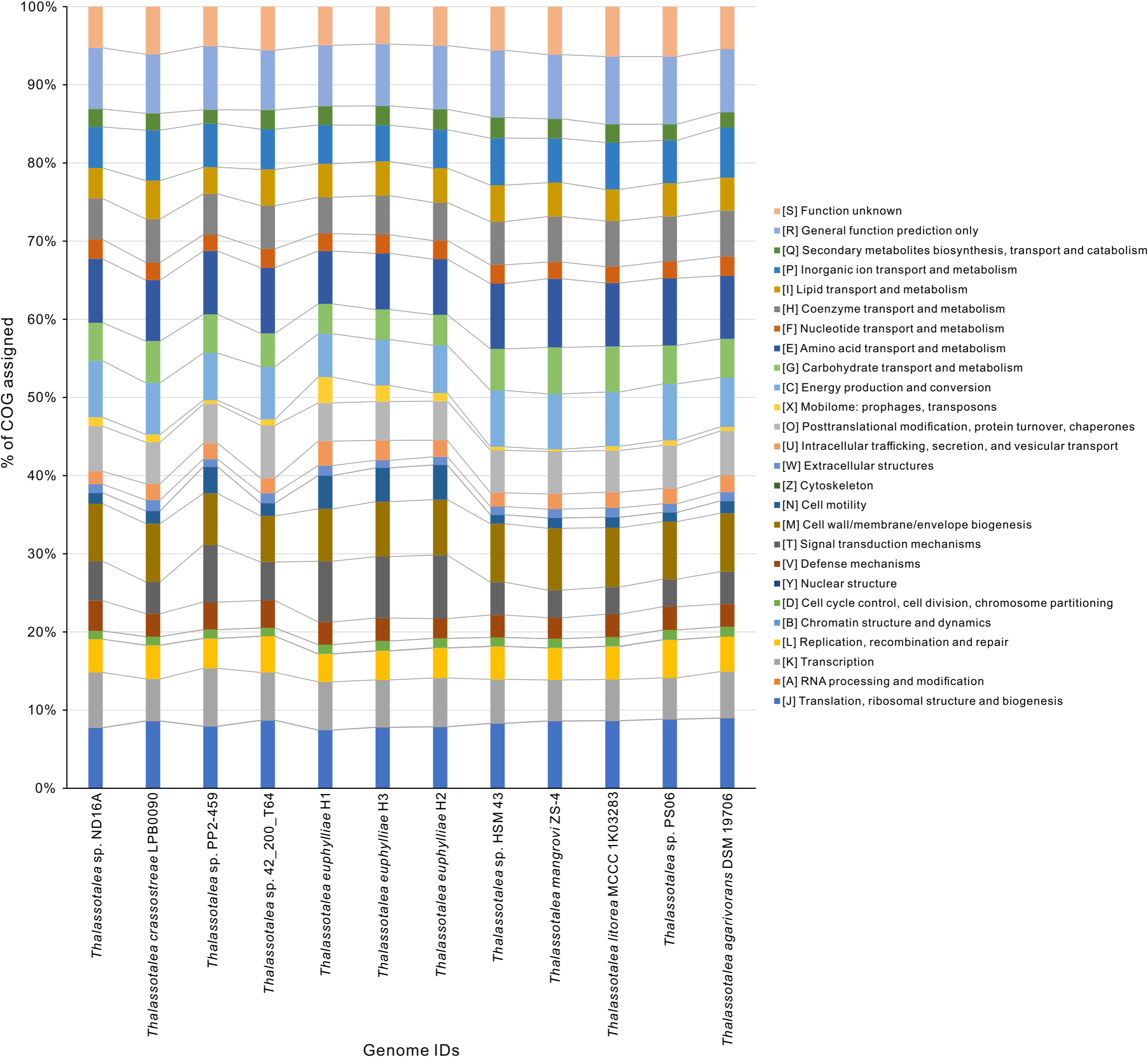

Supplement: Supplementary file 1 [file microorganisms-08-01412-s001.zip › supplementary/FigS1-thal-COG_rev.pdf]

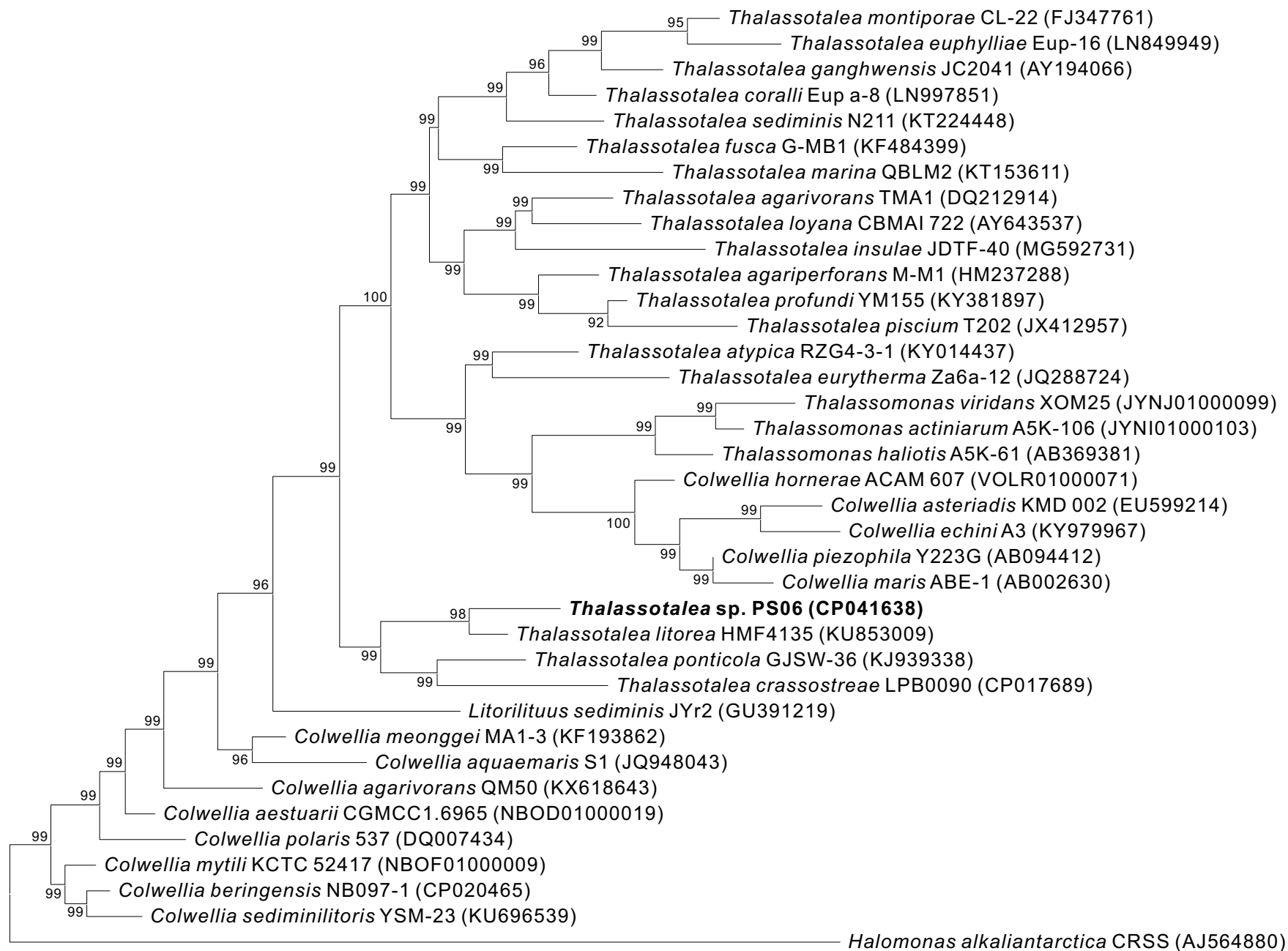

0.02

Supplement: Supplementary file 1 [file microorganisms-08-01412-s001.zip › supplementary/FigS2-ML-16Stree.pdf]
